# *Notes from the Field:* Acute Poisonings from a Synthetic Cannabinoid Sold as Cannabidiol — Utah, 2017–2018

**DOI:** 10.15585/mmwr.mm6720a5

**Published:** 2018-05-25

**Authors:** Roberta Z. Horth, Barbara Crouch, B. Zane Horowitz, Amelia Prebish, Matthew Slawson, Jennifer McNair, Chris Elsholz, Stephen Gilley, Jenny Robertson, Ilene Risk, Mary Hill, Linnea Fletcher, Wei Hou, Dallin Peterson, Karlee Adams, Dagmar Vitek, Allyn Nakashima, Angela Dunn

**Affiliations:** ^1^Epidemic Intelligence Service, CDC; ^2^Utah Department of Health, Salt Lake City, Utah; ^3^Utah Poison Control Center, Salt Lake City, Utah; ^4^Utah Department of Health, Public Health Laboratory, Taylorsville, Utah; ^5^Utah Department of Public Safety, Bureau of Forensic Services, Taylorsville, Utah; ^6^Utah State Bureau of Investigation, Salt Lake City, Utah; ^7^Utah Department of Public Safety, Statewide Information and Analysis Center, Sandy, Utah; ^8^Salt Lake County Health Department, Salt Lake City, Utah; ^9^Utah County Health Department, Provo, Utah.

On December 8, 2017, the Utah Poison Control Center (UPCC) notified the Utah Department of Health (UDOH) of reports of emergency department visits associated with reported exposure to products labeled as CBD (cannabidiol), a nonpsychoactive compound derived from *Cannabis sativa,* the marijuana plant. Five patients experienced adverse reactions, including altered mental status, seizures, confusion, loss of consciousness, and hallucinations. These reactions were inconsistent with known CBD effects (*1*), which prompted concern for potential adulteration with a synthetic cannabinoid (*2*). CBD is being studied as a treatment for several health conditions* (*3*); however, the Food and Drug Administration has not approved any CBD product for the treatment of any condition, and the U.S. Department of Justice Drug Enforcement Administration considers CBD as a Schedule I drug.^†^ Sale of CBD is currently illegal in Utah, although CBD is readily available online and in shops.

State and federal health and law enforcement officials established a task force on December 11 to investigate cases and identify the source product. A suspected case was defined as the occurrence after October 1, 2017, of adverse reactions inconsistent with known CBD exposures after ingestion, inhalation, or sublingual consumption of a product labeled as CBD or hemp oil. Hospitals and law enforcement agencies or persons experiencing CBD-associated reactions were asked to report any CBD-associated cases to UPCC. Concomitantly, public health investigators searched UPCC’s database and Utah’s Syndromic Surveillance system as part of CDC's National Syndromic Surveillance Program for CBD-related events.^§^ UDOH interviewed patients by telephone, using a survey adapted from a synthetic cannabinoid investigation (*4*). Available blood and urine obtained at emergency departments and product samples obtained from patients were submitted for chemical analysis using liquid chromatography and tandem mass spectrometry at the Utah Public Health Laboratory and the Utah Department of Public Safety crime laboratory.

By the end of January 2018, suspected cases were identified in 52 persons. Nine product samples (including one unopened product purchased by investigators from a store and brand reported by a patient) were found to contain a synthetic cannabinoid, 4-cyano CUMYL-BUTINACA (4-CCB), but no CBD.^¶^ Eight of the tested products were branded as “Yolo CBD oil” and indicated no information about the manufacturer or ingredients. Blood samples from four of five persons were positive for 4-CCB. Press releases were distributed to media outlets December 19–21, 2017, with a warning regarding the dangers of using the counterfeit product; information with a description of the product and associated symptoms was disseminated to health care providers and law enforcement. The number of reported cases peaked during this outreach and dropped shortly thereafter. Thirty-four suspected cases were reclassified as confirmed if the person reported use of a Yolo product or laboratory testing found 4-CCB. Approximately one quarter of persons were aged <18 years, nearly three fourths had vaped the CBD product, and approximately 60% were seen at an emergency department (Table). The top three symptoms experienced were altered mental status, nausea or vomiting, and seizures or shaking. Rapid identification and a coordinated response among state and local agencies contributed to control of the outbreak. This investigation highlights the hazards of consuming unregulated products labeled as CBD. States could consider regulating products labeled as CBD and establishing surveillance systems for illness associated with products labeled as CBD to minimize the risk for recurrences of this emerging public health threat (*5*).

**TABLE:** Characteristics of suspected or confirmed cases of poisoning associated with counterfeit cannabidiol products (N = 52) — Utah, 2017–2018

Characteristic	No. (%)
**Age group (yrs)**	
≥18	28 (53.8)
<18	15 (28.8)
Unknown	9 (17.4)
**Sex**	
Male	31 (59.6)
Female	14 (26.9)
Unknown	7 (13.5)
**County**	
Salt Lake	33 (63.5)
Utah	15 (28.8)
Tooele	3 (5.8)
Weber	1 (1.9)
Medical history*	
Mental health treatment	10 (19.2)
Drug abuse	4 (7.7)
Seizures	1 (1.9)
**Product brand**	
Yolo	33 (63.5)
Other	10 (19.2)
Unknown	9 (17.3)
**Source of purchase**	
Smoke shop	34 (65.4)
Friend	8 (15.4)
Unknown	10 (19.2)
**Reason for use**	
Recreational	35 (67.3)
Medicinal^†^	15 (28.8)
Other	2 (3.8)
**Method of use**	
Vape	38 (73.1)
Sublingual	9 (17.3)
Other	2 (3.8)
Unknown	3 (5.8)
**Seen at an emergency department **	
Yes	31 (59.6)
No or unknown	21 (40.4)
Adverse reactions*	
Altered mental status	43 (82.7)
Nausea or vomiting	26 (50.0)
Seizures or shaking	19 (36.5)
Anxiety	14 (26.9)
Unconsciousness	13 (25.0)
Hallucinations	12 (23.1)
Confusion	10 (19.2)
Dizziness	8 (15.4)
Median time to reaction onset after use, minutes (IQR)	35^§^ (1; 1–5)
Median duration of adverse reaction, minutes (IQR)	27^§^ (72; 5–72)

**Abbreviation:** IQR = interquartile range.

* Multiple responses possible.

^†^ Self-reported medicinal use.

^§^ Number for whom information was available.

## Footnotes

* CBD is used in treating spasticity from multiple sclerosis and Dravet syndrome, a severe form of childhood epilepsy, for which it has shown efficacy.

† A Schedule I drug, defined by the U.S. Department of Justice Drug Enforcement Administration, is a drug with no currently accepted medical use and a high potential for abuse.

§ The compound 4-CCB has been identified in Europe since 2016 when samples were intercepted as synthetic cannabinoids; 4-CCB is chemically related to other indazole-based synthetic cannabinoids, known as NACA derivatives, which are found in other synthetic cannabinoid clusters reported in the United States.

¶ Search terms included CBD-associated slang and brands. Search terms excluded symptoms because they were insufficiently specific.
